# De-Prescribing of Psychotropic Medications in the Adult Population with Intellectual Disabilities: A Commentary

**DOI:** 10.3390/pharmacy6020028

**Published:** 2018-03-30

**Authors:** Bernadette Flood

**Affiliations:** Daughters of Charity Disability Support Services, D15 DH6F, Dublin, Ireland; bernadette.flood@docservice.ie

**Keywords:** intellectual disabilities, psychotropic, pharmacist, de-prescribing, medicines optimization, vulnerable population, risk

## Abstract

The population with intellectual disabilities is one of the most vulnerable groups in society. Medication use is the main therapeutic intervention in this population and psychotropic medications can be prescribed for mental health conditions and for challenging behaviors. Clinical experience of prescribers and pharmacists working with people with intellectual disabilities suggests that reducing or stopping psychotropic medication is not always straightforward. What is required is rational, rather than rationed, prescribing of psychotropic medications. Concerns of clinicians working with people with intellectual disabilities and both formal and informal carers can result in maintenance of the ‘status quo.’ Setting-related, carer-related and staff-related factors play an important role in the real world of people with intellectual disabilities. Optimizing medication regimens in the adult population with intellectual disabilities is complicated but it is recognized that efforts to improve the current state of medication utilization are required for many individuals with intellectual disabilities. Pharmacists have a responsibility to include the person and/or their carer in their efforts to promote optimization of psychotropic medication use in environment in which the person lives.

## 1. Background

Intellectual disability is a disability characterized by significant limitations in both intellectual functioning and in adaptive behavior, which covers many everyday social and practical skills. This disability originates before the age of 18 [1]. Generally, an IQ test score of around 70 or as high as 75 indicates a limitation in intellectual functioning. Intellectual disability is the preferred term to describe those who were diagnosed previously with mental retardation. The term learning disability is used as the official term for intellectual disability in England.

Advances in the treatment of medical conditions mean that people with intellectual disabilities are living with multiple co-morbidities for longer than in the past. Medication use is the main therapeutic intervention in this vulnerable population and psychotropic medications can be prescribed for diagnosed mental health conditions and for challenging behaviors. The DC-LD (Diagnostic Criteria for Psychiatric Disorders for Use with Adults with Learning Disabilities; Royal College of Psychiatrists, 2001) classificatory system (designed specifically for people with intellectual disabilities and to complement ICD-10) describes a person’s mental health on axes and levels: severity of intellectual disabilities, causes of intellectual disabilities, psychiatric disorders including developmental disorders, psychiatric illness, personality disorders, problem behaviors and others [2]. The Royal College of Psychiatrists (2016) defines challenging behavior as follows: ‘*Behavior can be described as challenging when it is of such an intensity, frequency or duration as to threaten the quality of life and/or the physical safety of the individual or others and is likely to lead to responses that are restrictive, aversive or result in exclusion*’ [3]. (There are a variety of terms used to describe challenging behaviors/behavior disorders/behaviors that challenge/problem behaviors. In this commentary article the term behaviors that challenge will be used for clarity.) 

The population with intellectual disabilities experience a different pattern of morbidity and mortality to the general population [4]. Members of this population group present with different patterns of illness and die younger. Mortality among adults with intellectual disabilities is significantly raised in comparison with the general population, with more than a third of deaths potentially amenable to health care interventions. This inequality in mortality suggests the need to improve access to and quality of, health care among people with intellectual disabilities [5].

Pharmacists and others have a duty to ensure that psychotropic medications are used appropriately to facilitate positive health outcomes in the adult population with intellectual disabilities and, behaviors that challenge to minimize harm to patients, improve quality of life and help reduce health inequality and inequity. The quality of prescribing and de-prescribing of psychotropic medication will be of interest to adults with intellectual disabilities and their carers. 

Patient-important outcomes such as quality of life or patient satisfaction are of particular importance in the population with intellectual-disabilities and for those who provide formal and informal care. This is similar to many older adults (and patients at end-of-life), who value quality of life and decreased burden of care over risk reduction or prolonging life [6].

Points to be considered by pharmacists (and others) supporting adults people with intellectual disabilities

- Mental illness is common in people with intellectual disabilities. They may also have physical health problems which can affect their mental health.
- Difficulties in communication can contribute to mental health problems being overlooked. These may present with changes in behavior and behaviors that present challenges to carers, professionals and service providers.
- Psychological management is usually preferable to prescribing psychotropic drugs. Behavioral approaches are the most appropriate way to manage behaviors that challenge.
- If a drug is considered, prescribers should complete a thorough diagnostic assessment and consider comorbidities before prescribing.
- Where possible psychotropic medications with the highest cardio-metabolic burden should be avoided. The minimum effective dose and treatment length should be prescribed and drug efficacy and adverse effects monitored regularly.

## 2. Behaviors that Challenge

Many people with intellectual disabilities have limited intellectual capacities and social-adaptive abilities which impacts on their reasoning and communication skills. Members of the population with intellectual disabilities have a higher risk of developing mental health difficulties or challenging behavior than the general population. The accepted range for prevalence of behavior that challenges is approximately 5 to 15% of people with an intellectual-disability who are known to services [7]. Forms of behaviors that challenge consist of externalizing behaviors such as aggression and destruction as well as internalizing behaviors such as social withdrawal and self-injurious behavior. Such behavior can significantly limit their quality of life and contribute to reduced participation in community and social activities. Behaviors that challenge also negatively impact on carers who may be informal, unpaid family members or paid support staff (with varying levels of training), who experience higher levels of stress, burnout and mental health problems when working with people with challenging behaviors [8].

There is a wide literature describing behaviors that challenge in difference population groups [9]. People with intellectual disabilities can become distressed and exhibit behaviors that challenge, when there is a mismatch between their personal abilities and resilience, their weaknesses and vulnerabilities and their living, social and physical environments, that is, their real-world environment. Some behaviors that challenge can also be an indication of unmet needs such as comfort, stimulation or safety. In these situations, a mental health illness or ‘behaviors that challenge’ can be diagnosed. Other features particular to this vulnerable population, that may contribute to behaviors that challenge are increased risks of traumatic or negative life histories, impoverished social networks, lack of meaningful activity or employment, sensory or health problems and genetic syndromes. 

The prescribing and administration of psychotropic medication is one recognized response to the development of behaviors that challenge in members of this vulnerable population. There is however insufficient evidence to support the use of psychotropic medications for behaviors that challenge [10,11]. These medications should be avoided unless the behavior is severe and non-responsive to other treatments as many have potentially serious side-effects. The initiation of psychotropic medications for a person with an intellectual disability, whether from primary or secondary care, should be by a prescriber who is competent in the care of people with intellectual disabilities [12]. There is a role for competent and specialist intellectual disability pharmacists to support prescribers, patients with intellectual disabilities and carers [13]. The limited evidence available in the literature suggests that pharmacists can make positive interventions in relation to the quality of the medication use process, in collaboration with other healthcare professionals, carers and patients with intellectual disabilities [14].

## 3. Psychotropic Medications

There are many complexities in prescribing, dispensing and administering psychotropic medication for adults with intellectual disabilities. Many adults may lack capacity to consent to treatment and may display a greater sensitivity to drug related side effects and adverse reactions. Concern has grown in the health and social care communities that many people with intellectual disabilities are receiving psychotropic medication when they do not have a diagnosed mental illness. The concerns relate in particular to inappropriate use of antipsychotic medications in people with intellectual disabilities for the treatment of behaviors that challenge. Two large pharmaco-epidemiological studies in UK and England have indicated a marked disassociation between rates of psychotropic prescription in people with intellectual disabilities and recording of underlying mental illness for which they are indicated [15,16]. Efforts have been made to determine the Drug Burden in older people with intellectual disabilities [17]. The Winterbourne View abuse scandal laid bare inappropriate use of psychotropic drugs [18]. The Learning Disabilities Census in England [19] showed that 72% of patients with intellectual disabilities in hospitals had received antipsychotic medication either regularly or as required in the 28 days prior to census collection. 

Pharmacists and others have become involved in efforts to reduce the rate of psychotropic medication prescribing in this vulnerable population. One aim of the NHS England STOMP (*Stopping Over-Medication of People with a Learning Disability*) [20] campaign is to ensure people with intellectual disabilities get the right medicine if they need it. This campaign advises that medication is regularly reviewed and that health professionals involve people and their families/carers in decisions about their medicines and other supports. In Australia pharmacists have expressed frustration about general practitioners disregarding their recommendations to de-prescribe anticholinergic and sedative medications [21]. General practitioners considered that de-prescribing of these medications should be undertaken by specialists.

## 4. Medicines Optimization

The term de-prescribing was used in the English language health literature in 2003 in an Australian hospital pharmacy journal [22] in an article titled, ‘*De-prescribing: achieving better health outcomes for older people through reducing medications*.’ The article outlined the principles of de-prescribing, with emphasis on reviewing all current medications, identifying medications to be ceased, substituted or reduced, planning a de-prescribing regimen in partnership with the patient and frequently reviewing and supporting the patient. 

Before considering de-prescribing, it is important for pharmacists and others to be aware of and recognize any health and well-being gaps, care and quality gaps and funding and efficiency gaps that exist in the health and social care provided to people with intellectual disabilities. Clinical experience of prescribers and pharmacists working with people with intellectual disabilities suggests that reducing or stopping psychotropic medication is not always straightforward. Some prescribers are reluctant to consider changes to medication regimen that might have been unchanged for years and where it has become difficult to judge the (positive or negative) impact of treatment [23]. General practitioners caring for people with intellectual disabilities (who may be recently discharged from long term care) may not feel competent to initiate medication changes and specialist psychiatrists with knowledge of this complex population group might be difficult to access. Incomplete notes or staff changes may result in knowledge of the original indication for medication or previous attempts to discontinue medication being forgotten. This may happen particularly where people have moved between care settings, transitioned from child to adult services, or returned home from specialist placements away from their home area. Formal and informal carers and professionals may have limited confidence in their ability to obtain crisis support or respite services if the need arises. In addition, people with intellectual disabilities and their carers frequently feel excluded from psychotropic medication decisions, deprived of options and can find it difficult to ask for more information or to challenge decisions [24]. Such ‘real world’ concerns will result in maintenance of the ‘status quo.’

Pharmacists and others must not focus only on medication reduction. People with intellectual disabilities must not be deprived of the undoubted benefit that some individuals with intellectual disabilities obtain from psychotropic medication. What is required in this population group is rational, rather than rationed, prescribing of psychotropic medications [23]. The term medicines optimization is preferred to reflect the broader context in which prescribing decisions in the population with intellectual disabilities are made. The Royal Pharmaceutical Society (2013) sets out four important principles of “medicines optimization” [25]: aim to understand the patient’s experience; evidence based choice of medicines; ensure medicines use is as safe as possible; make medicines optimization part of routine practice. These simple principles have particular relevance when providing pharmaceutical care to people with intellectual disabilities and behaviors that challenge.

Optimizing medication regimen in the population with intellectual disabilities is complicated but it is recognized that efforts to improve the current state of medication utilization are required. A systematic review of the literature on reducing or discontinuing antipsychotic medication for challenging behavior found that while a significant proportion of people with intellectual disabilities could have their antipsychotic medication reduced or stopped, a roughly equal number suffered an array of adverse effects [26]. In some cases, this required medication to be re-prescribed at higher doses. The authors noted that decisions to reduce or stop psychotropic medication must therefore be taken on an individual basis. 

Prescribing, de-prescribing and/or optimizing psychotropic medication use in the population with intellectual disabilities does not only involve prescribers. Setting-related and staff-related factors play a prominent role. These include setting policies regarding restrictive measures, attitudes, knowledge and beliefs of clients, family and staff concerning the effects of antipsychotics in people with intellectual disabilities and attitudes of nursing staff towards challenging behavior of the people in their care. The availability and effectiveness of alternative, pro-active interventions for complex presentations and the provision of good quality care and support is crucial. Studies have shown up to a four-fold difference in rates of antipsychotic prescribing for challenging behavior between people with different living environments, despite there being no significant difference in the prevalence of behavior disorder [27].

De-prescribing of antipsychotic medications through reduction or discontinuation may not be successful. The following reasons have been suggested for this failure [28]. 1. There is the influence of the subjective interpretation of behavioral symptoms by caregivers and family; 2. Some people with intellectual disabilities will benefit from antipsychotic treatment and 3. When antipsychotics are withdrawn after long-term treatment, withdrawal symptoms might occur.

### Guidelines

Pharmacists and others need to think outside the box to find practical ways of improving the lives of people with intellectual disabilities and complex additional needs, such as behaviors that challenge. Psychotropic medication is only one piece of the puzzle and cannot be seen in isolation. Few guidelines exist in the literature to help practitioners make decisions about the health of their adult patients with intellectual disabilities. Most strategies and guidelines and so forth are based on the health needs of the general population. As the pattern of health need and causes of death differ for people with intellectual disabilities, the use of guidelines/tools/formularies and so forth developed in population groups that have not considered the population with intellectual may widen the health inequity and inequality gaps. For example, the Beers list [29] and STOPP [30] criteria provide explicit lists of medications considered inappropriate in older adults. Before they are considered for use in the population with intellectual/learning disabilities and behaviors that challenge, the appropriateness of these tools in an individual with an intellectual disability and their living and care environment need to be considered. Any tool used in the de-prescribing process or to consider the appropriateness or otherwise of psychotropic medication should be validated in the population with intellectual. There is a group of people with intellectual disabilities which displays behavioral deterioration on antipsychotic reduction that prevents discontinuation. As predictors of poor response have not been reliably identified [31], care is required by pharmacists and others involved in de prescribing.

The Royal College of Psychiatrist’s Good prescribing practice guidance, aimed at healthcare clinicians, is among the few guidelines focused on this population and proposes standards for improving clinical practice in the area of intellectual disability care It covers the prescription of any psychotropic medication, including antipsychotics, antidepressants, anxiolytics and mood stabilizers and sets out a framework for clinicians on how to rationalize their prescribing practice and, where appropriate, taper and stop psychotropic drugs. The guideline recommends that all initiations of psychotropic drugs for people with intellectual disability, whether from primary or secondary care, should be by a prescriber who is competent in the care of people with intellectual disabilities.

## 5. Considerations When Prescribing and De-Prescribing

The population with intellectual disabilities are vulnerable in the prescribing and the de-prescribing process [32]. They and their carers may not be involved in either process and may not have been provided with relevant accessible patient centered information. The provision of information through training in a format that was understandable by a small group of people with intellectual disabilities was shown to increase their knowledge of medication [33]. Easy Read leaflets are available that provide people with an intellectual disability with information about medicines that are used for behaviors that challenge [31].

Adults with intellectual disabilities use multiple medications including psychotropic medications and may have been taking them for many years. The side effects of antipsychotic medication should be reviewed at least once a year and this review should include assessment for the presence of extrapyramidal side effects and screening for the four aspects of the metabolic syndrome: measures of blood pressure, obesity, glycemic control and plasma lipids [24]. De-prescribing of psychotropic medications should be considered if treatment is ineffective, there are unacceptable adverse effects, discontinuation is requested, symptoms have resolved or the drug is no longer required. However, care is required when de-prescribing many psychotropic medications in this population group.

Ten principles of good de-prescribing during medication review in the population with intellectual disabilities, based on the British Pharmacological Society’s Principles for Good Prescribing 2010 [34]

1. Be clear about the reasons for de-prescribing psychotropic medication.
2. Take into account the patient’s medication history before de-prescribing.
3. Take into account other factors that might alter the benefits and risks of de-prescribing psychotropic medication.
4. Take into account the patient’s/carer’s/families/advocates ideas, concerns and expectations. Share information about the benefits and harms of different options and allow patients/carers to clarify what is important to them about these options.
5. Ensure all medicines are effective, safe, cost-effective, in appropriate form and individualized for the patient with intellectual disability, behaviors that challenge and other conditions such as dysphagia, autism.
6. Adhere to national guidelines and local formularies where appropriate. Use caution where the population with intellectual disabilities have not been considered in the guideline/formulary development process.
7. Write unambiguous accurate documentation detailing reason for de-prescribing psychotropic medications (or other medications).
8. Monitor and document the beneficial and adverse effects of de-prescribing psychotropic medicines and any effects on behavior.
9. Communicate and document all de-prescribing decisions and the reasons for them and ensure information communicated to appropriate personnel such as GP, pharmacist, psychiatrist, epileptologist, carer and patient.
10. De-prescribe psychotropic medications within the limitations of your knowledge, skills and experience of the population with intellectual disabilities and behavior disorders.

## 6. Take Home Message

The adult population with intellectual disabilities is one of the most vulnerable groups in society. The prescribing and de-prescribing of psychotropic medications may put them at risk of adverse events and poor quality care. Doctors have a particular responsibility to ensure that they have fully assessed a person’s potential to benefit from medication before they prescribe. They must also check that the anticipated benefits have occurred after they have prescribed [12]. Pharmacists have a responsibility to include the person with an intellectual disability and/or their carer in their efforts to promote optimization of psychotropic medication use. 

## Glossary

Behavior that challenges	behavior that provides challenges to carers, professional staff, management.
Carer	someone who takes care of a person who needs regular assistance because of an illness, disability or the inability to do some everyday tasks on their own. Care may be provided on a formal (paid) or an informal (unpaid) basis. Care may be regulated or unregulated.
Specialist	a person who concentrates primarily on a particular subject or activity; a person highly skilled (through education, experience or interest) in a specific and restricted field.
